# Differences in Time until Dispersal between Cryptic Species of a Marine Nematode Species Complex

**DOI:** 10.1371/journal.pone.0042674

**Published:** 2012-08-02

**Authors:** Nele De Meester, Sofie Derycke, Tom Moens

**Affiliations:** 1 Marine Biology Unit, Department of Biology, Ghent University, Ghent, Belgium; 2 Center for Molecular Phylogeny and Evolution, Ghent University, Ghent, Belgium; University College Dublin, Ireland

## Abstract

Co-occurrence of closely related species may be achieved in environments with fluctuating dynamics, where competitively inferior species can avoid competition through dispersal. Here we present an experiment in which we compared active dispersal abilities (time until first dispersal, number and gender of dispersive adults, and nematode densities at time of dispersal) in *Litoditis marina*, a common bacterivorous nematode species complex comprising four often co-occurring cryptic species, Pm I, II, III, and IV, as a function of salinity and food distribution. The experiment was conducted in microcosms consisting of an inoculation plate, connection tube, and dispersal plate. Results show species-specific dispersal abilities with Pm I dispersing almost one week later than Pm III. The number of dispersive adults at time of first dispersal was species-specific, with one dispersive female in Pm I and Pm III and a higher, gender-balanced, number in Pm II and Pm IV. Food distribution affected dispersal: in absence of food in the inoculation plate, all species dispersed after ca four days. When food was available Pm I dispersed later, and at the same time and densities irrespective of food conditions in the dispersal plate (food vs no food), suggesting density-dependent dispersal. Pm III dispersed faster and at a lower population density. Salinity affected dispersal, with slower dispersal at higher salinity. These results suggest that active dispersal in *Litoditis marina* is common, density-dependent, and with species, gender- and environment-specific dispersal abilities. These differences can lead to differential responses under suboptimal conditions and may help to explain temporary coexistence at local scales.

## Introduction

Biodiversity in many ecosystems appears significantly higher than previously thought due to cryptic genetic diversity which underlies a broad range of morphospecies [1], [2]. Despite increasing documentation of cryptic diversity, knowledge about the ecology of cryptic species remains very scant (e.g. [3], [4], [5], [6], [7]). Morphologically highly similar species may show high functional similarity and niche overlap [8], [9], [10] which seems at odds with traditional competition theory [11], [12], [13].

Coexistence of closely related species can be achieved in environments with fluctuating dynamics in time or space (e.g. presence of other species, food distribution). Here, competitively inferior species may persist because they are temporarily favoured by specific conditions [14]. Alternatively, species that are sufficiently motile can move to suitable patches and thus avoid competition [15]. In this way, they can at least temporarily achieve some form of coexistence but escape from it through small-scale dispersal when competitive pressure becomes too strong. This movement of individuals away from their natal environment is dispersal and can lead to gene flow over different spatial scales [16], [17]. Dispersal is a process, triggered partly by the intrinsic condition of organisms, such as gender, competitive ability, genetic variability and species identity [18], [19], [20], [21], [22], and partly by environmental conditions, such as habitat and food quality, population density and intraspecific interactions [23], [24], [25], [26].

In contrast with most larger marine benthic vertebrates having at least one life stage in which dispersal occurs on a specific spatial scale [16], most meiobenthic species (nematodes and other small metazoans in the size range of 0.04 to 2 mm [27]) lack a pelagic stage and have long been considered poor dispersers due to their small size and poor swimming ability [28]. Nematodes are the most abundant meiofauna in marine sediments [29], [30] and have a high species diversity at both global and local scales [31]. They can passively disperse following erosion from sediments or through rafting on algae ([32]), but they can also actively enter the water column [19], which may facilitate both small-scale active dispersal as well as larger-scale passive dispersal [33] [34]. They can at least partly control their settlement back to the sediment [35]. In addition, they migrate laterally through sediments [19], [20], but the rates and distances over which nematodes actively disperse and the extrinsic and intrinsic drivers of dispersal remain poorly known. Salinity effects, for instance, have not been tested before, probably because the effect of salinity variation has mostly been considered on a broader geographical scale. However, diurnal [36] and seasonal variations [37] in salinity also occur, which may affect small-scale dispersal of meiofauna in a direct or indirect way [18].

*Litoditis marina* [38] is a common bacterivorous nematode associated with decomposing macroalgae in the littoral zone of coastal and estuarine environments [39], [40]. Several cryptic species have been found within this morphospecies, formerly known as *Rhabditis marina* or *Pellioditis marina* [41], four (Pm I, Pm II, Pm III and Pm IV) of which frequently occur along the south-western coast and estuaries of The Netherlands [41]. Moreover, it is common to find two or three of these cryptic species co-occurring [42]. These species show concordant molecular divergences at nuclear and mitochondrial loci but lack single distinctive morphological differences [43], [44], and crossbreeding between them does not occur [44]. All species are gonochoristic; parthenogenesis has hitherto not been observed [37]. Females of Pm I and IV largely reproduce through ovovivipary, whereas Pm II and Pm III use ovipary They produce several tens up to 600 progeny per female [45], [46], [47], the vast majority of which are released during the first few days following maturation to adults. All four species have minimal generation times of ca. 4 days at temperatures around 20°C, salinities between 15 and 30, and sufficient food availability (De Meester et al., unpublished data). Both geographical and seasonal variation in abundance and dominance of these cryptic species occurs [42] and may be linked to environmental variation (e.g. salinity). Recent laboratory experiments have also demonstrated that salinity affects the outcome of competitive and facilitative interactions between these cryptic species, with competition being more pronounced at lower salinity and Pm IV and Pm II being competitively inferior to Pm I and Pm III [4].

In the present study, we tested if differences in dispersal abilities between the four different cryptic species of *L. marina* exist and if these differences are gender- and environment-specific. In a first experiment the effects of food distribution on the dispersal ability of cryptic species are tested. If active dispersal exists in the cryptic species, we expect that it will occur more when food is limited in the source patch but still available in nearby patches. In a second experiment dispersal abilities of the four cryptic species were tested at different salinities. A previous experiment already showed that population growth and competitive ability of two of the four cryptic species (Pm III and Pm IV) differed between two salinities [4]. At the lower salinity, population growth rate was higher, suggesting that this salinity was more favourable. We thus expected that this faster population growth would result in more pronounced intraspecific competition, leading to faster dispersal at the lower salinity [48], [49]. These experiments will yield insight in extrinsic (salinity and food distribution) and intrinsic (species identity and gender) factor-dependent dispersal. Investigating the dispersal abilities of species is crucial to understand the highly dynamic patterns and the ecology of meiobenthic communities [50] and the resilience of populations under fluctuating environmental conditions [51].

## Materials and Methods

### Nematode cultures

Monospecific cultures of the four different cryptic species were raised from one single gravid female per species and maintained on sloppy (1%) nutrient∶bacto agar media [52] (temperature of 20°C; salinity of 25) with unidentified bacteria from their habitat as food. Species identity and monospecificity of stock cultures were tested shortly after their initiation and on regular moments thereafter on several individuals with a species-specific qPCR assay using ITS sequences [53]. Nematodes for the experiments were harvested from these stock cultures in exponential growth phase.

### Dispersal experiments

**Figure 1:**
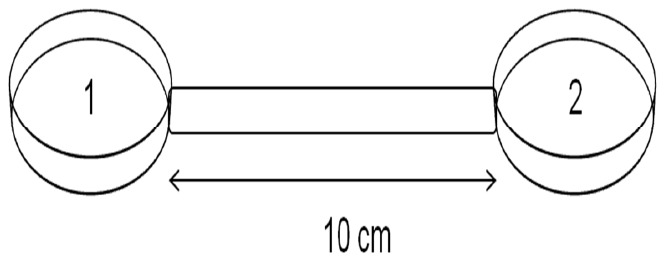
Design of the dispersal microcosms with plate 1 being the inoculation plate and plate 2 the dispersal plate. Dispersal ability was scored when nematodes first arrived at plate 2.

Dispersal abilities of the cryptic species were measured as time until the first effective dispersal event. Dispersal was considered effective if it was followed by reproduction in the dispersal plate, regardless of whether the individual was already gravid before the dispersal event. To study the differences in time until dispersal between the four cryptic species, specially designed dispersal plates were used (Fig. 1). These plates consist of two Petri dishes (each 5 cm i.d.; an ‘inoculation’ plate and a ‘dispersal’ plate, respectively) connected by a tube (1 cm i.d and 10 cm length). The length of this test tube was based on results of a preliminary test with tubes of various lengths. Considerably shorter tube lengths resulted in almost instantaneous migration to the dispersal plate, through random movement and/or through direct chemotaxis to food on the dispersal plate. Longer tube lengths (≥15 cm) resulted in very slow dispersal irrespective of presence of food in the dispersal plate. The substratum in the plates was provided as 60 mL of a 1.5% bacto agar medium prepared with artificial seawater [54]. The agar was spread equally over the two different plates and the connection tube taking care that the surface was at the same level and continuous in both plates and connection tube. The relatively high concentration of the agar (1.5%) hampers burrowing of nematodes into the agar and thus restricts their movement to the agar surface, which greatly facilitates observations. The pH of the agar medium was buffered at 7.5–8 with TRIS-HCl in a final concentration of 5 mM. The addition of the buffer increases the initial salinity by ca 1.2 units. Two sets of dispersal experiments were performed, the first focusing on the role of food availability in the dispersal and inoculation plates, the second focusing on the effect of salinity on dispersal.

#### (a) Food distribution experiment

The experiment was started by manually picking up five adult males and five adult females from the stock cultures of a single cryptic species. Before placing the organisms randomly in the inoculation plate, they were bathed in clean artificial seawater (salinity of 25) for 2 h to remove most adhering bacteria. For every cryptic species, four different treatments were used. In the ‘B’ treatment food was added to both plates (inoculation and dispersal plate); in the ‘I’ treatment food was only added to the inoculation plate and not to the dispersal plate; in the ‘D’ treatment food was only added to the dispersal plate, and in the ‘N’ treatment food was absent from both plates. Food consisted of frozen-and-thawed *Escherichia coli* (strain K12) *and was added ev*ery eighth da*y*, well before food depletion occurred (50 µL of a suspension with a density of 3×10^9^ cells mL^−1^) [47]. This bacte^ri^al strai^n^ has been shown to be a suitable food source for cultures of these four cryptic species of *L. marina*. No food was ever ad*ded to th*e connection tube, but dispersing nematodes do carry bacteria on their cuticles and thus spread some food into the connection tube and dispersal plate. All plates contained agar with a salinity of 25 and were incubated in the dark at a constant temperature of 20°C. There were four independent replicates per treatment.

Population and dispersal dynamics were studied by counting adults and juveniles every day in both plates. The timing of the arrival of the first organism at the dispersal plate was recorded, as well as the life stage (adult or juvenile) and the effectiveness of the dispersal event. Observations on the organisms in the connection tube and on nematode tracks were made, to verify if organisms were moving from the inoculation to the dispersal plate and not in a random way. Moreover, the gender of the first dispersers was recorded for the B treatment. After 20 days, the experiment was stopped because the agar medium started to become liquid. By that time, dispersal had occurred in every replicate.

#### (b) Salinity experiment

Additional dispersal plates were started with food in both plates (treatment B, see above), but with agar medium with a salinity of 15 instead of 25. Methods and incubation conditions were the same as described for the food distribution experiment.

### Data analyses

Differences in the time until first effective dispersal between the cryptic species and between food treatments were tested in R [55] with a two-way ANOVA (species and food distribution as independent variables), as the assumptions for parametric tests were met. Abundances of adults, juveniles and total nematodes in the inoculation plate at the moment of first effective dispersal were also compared between the different species and food distributions by using a two-way ANOVA. A Tukey HSD test was used for posterior pair wise comparisons. A log transformation on the adult abundances in the dispersal plate at first dispersal event was used, as the data were not normally distributed. The analyses for the dispersive organisms were conducted with the data of the adults only. Juveniles were omitted from the analyses, as Pm I and Pm IV are ovoviviparous species and in this way it was not possible to determine whether the juveniles present in the dispersal plate were real dispersers or offspring of dispersed adults.

Differences in the time until first effective dispersal between the cryptic species and between salinities were also tested with ANOVA, and so were the abundances of adults, juveniles and total nematodes in the inoculation plate at the moment of dispersal, and the number of dispersive adults. When no significant interaction effects were found, one-way ANOVA or Kruskal-Wallis tests within one species were conducted to look for the effect of salinity within each species separately.

Differences in gender-specificity of dispersal between the cryptic species was tested by calculating the proportion of females at the first dispersal event and conducting a Kruskal-Wallis test with proportion of females as dependent variable and species as independent variable, as the assumptions for parametric tests were not met.

## Results

### Food effects on time until dispersal between the different cryptic species

**Figure 2:**
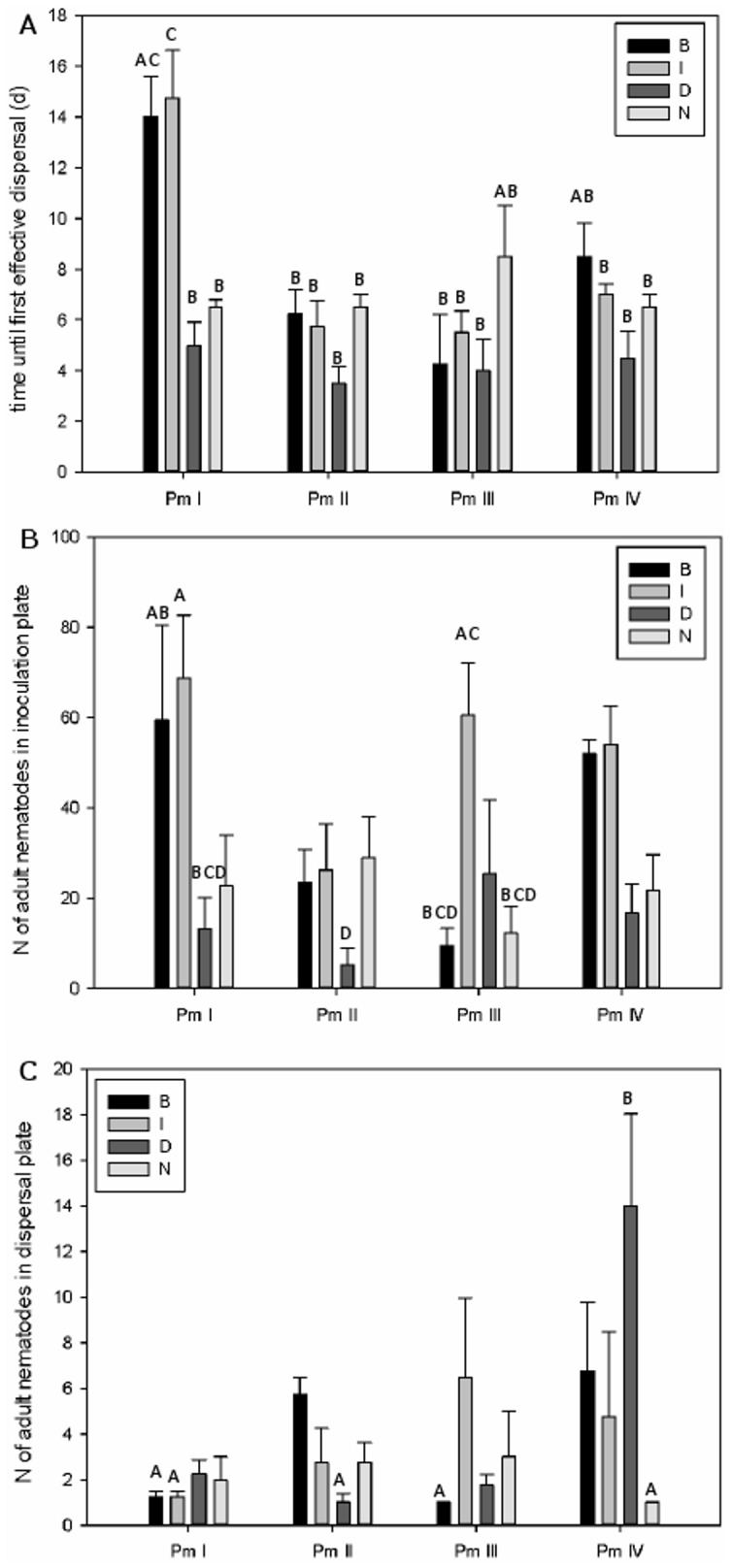
Effect of food distribution on dispersal abilities: (a) average time until first dispersal event (mean ± SE), (b) average number of adults in the inoculation plate (mean ± SE) at time of first dispersal event and (c) average number of adults in the dispersal plate (mean ± SE) at time of first dispersal for the four cryptic species of *L. marina* at four different food treatments. (B: food at inoculation and dispersal plate; I: only food in inoculation plate; D: only food in dispersal plate; N: no food in both plates) (letters above bars indicate pairwise significant differences; p<0.05; n = 64).

Time until first effective dispersal differed between the four cryptic species of *L. marina* (ANOVA, F_3,48_ = 13.56, P = 2.06e^−05^) and the distribution of food had a significant effect on this (F_3,48_ = 10.47, P = 1.56e^−06^). Moreover, an interaction effect between species and food distribution was found (F_9,48_ = 4.89, P = 0.00012). Food distribution had a pronounced effect on the time until dispersal of Pm I, with a significantly longer time until dispersal for the B and the I treatment (dispersal occurred respectively after 14.5±1.6 days and 14.8±1.9 days) compared with the D and N treatment (resp. average of 5±0.9 and 6.5±0.3 days until dispersal, fig. 2a). In the B and I treatment, Pm I also dispersed more slowly than the other species, except for Pm I in the B treatment compared with Pm IV in the B treatment and with Pm III in the N treatment (for B treatment: 6.3±1.0 days (Pm II), 4.3±2.0 days (Pm III); for I treatment: 5.8±1.0 days (Pm II), 5.5±0.9 days (Pm III) and 7.0±0.4 days (Pm IV); for N treatment: 6.5±0.5 days (Pm II) and 6.5±0.5 days (Pm IV)). Time until dispersal for the D treatment did not differ between the species and no differences between the different food treatments were found for the other species (Fig. 2a).

Total nematode density in the inoculation plate at the moment of first effective dispersal only differed between the different food distribution treatments (ANOVA, F_3,48_ = 11.40, P = 9.19e^−06^), with significant differences between the I treatment and the three other treatments. Dispersal occurred at the lowest nematode density for the D treatment (47.6±15.2 organisms over the four species), followed by the N treatment (114.7±26.5 organisms), the B treatment (119.1±29.8 organisms) and the I treatment (215.8±23.7 organisms). Food distribution had the same effect on juvenile and adult densities in the inoculation plate at first dispersal (resp. F_3,48_ = 9.63, P = 4.37e^−05^ and F_3,48_ = 10.36, P = 2.28e^−05^). In addition, adult numbers in the inoculation plate differed between species (F_3,48_ = 3.06, P = 0.037), and a significant interaction effect between food distribution and species was observed (F_9,48_ = 2.16, P = 0.042) (Fig. 2b), with lower adult numbers at time of first dispersal for Pm II in the D treatment (5.3±3.6 adults) compared with Pm I in the B and I treatment (resp. 59.5±20.9 adults and 68.8±10.2 adults) and Pm III in the I treatment (60.5±11.7 adults).

For number of dispersive adults at time of first effective dispersal the interaction between food distribution and species was significant (ANOVA on log-transformed data, F_9,48_ = 3.09, P = 0.0052). Significant differences between species were also found (F_3,48_ = 3.00, P = 0.039), mostly the result of a higher number of dispersive adults for Pm IV in the D treatment (14±4.02 adults) compared with Pm II in this treatment (1.0±0.5), Pm I in the B and I treatment (resp. 1.3±0.3 and 1.3±0.4 adults), Pm III in the B treatment (1.0±0.0 adults) and Pm IV in the N treatment (1.0±0.0 adults) (Fig. 2c).

### Salinity effects on time until dispersal between the different cryptic species

**Figure 3:**
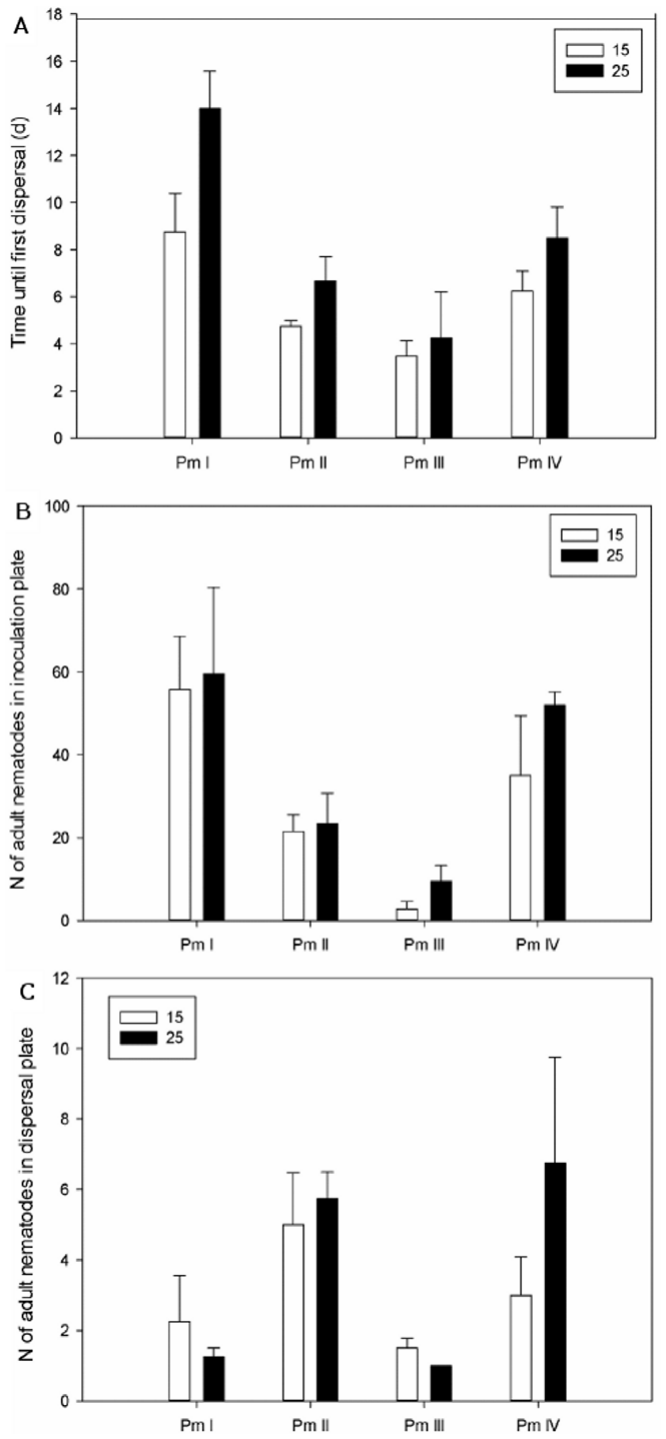
Effect of salinity on dispersal abilities: (a) average time until first dispersal event (mean ± SE), (b) average number of adults in the inoculation plate (mean ± SE) at time of first effective dispersal and (c) average number of adults in the dispersal plate at the first dispersal event (mean ± SE) for the four cryptic species of *L. marina* at two different salinities (no pairwise significant differences were found; p<0.05; n = 32).

Time until first effective dispersal was shorter at a lower salinity for all species (ANOVA, F_1,24_ = 7.32, P = 0.012), with an average of 5.8±1.1 days at a salinity of 15 compared to 8.4±2.1 days at a salinity of 25. Time until dispersal also differed between the four cryptic species over the two salinities (F_3,24_ = 12.9, P = 3.28e^−05^) with Pm I again being the slowest disperser. Dispersal in Pm I occurred only after 11.4±1.5 days (average over the two salinities) compared to 5.5±0.5 days in Pm II, 3.9±0.97 days in Pm III and 7.4±0.8 days in Pm IV (Fig. 3a). No interaction effect between species and salinities was found (F_3,24_ = 1.19, P = 0.33), indicating that the salinity effect was similar for all four cryptic species. However, no significant differences in time until first dispersal within species could be found (F_1,6_, all P>0.05).

Total numbers of organisms in the inoculation plate at time of first effective dispersal were lower at a salinity of 15 than at a salinity of 25 (77.3±15.3 vs. 126.7±16.1 organisms; ANOVA, F_1,24_ = 5.49, P = 0.028) over the four cryptic species. Total abundances at time of dispersal were also species-specific (F_3,24_ = 6.91, P = 0.0021), with Pm III dispersing at much lower total densities (28.1±12.0 organisms) compared with the three other species (resp. 139.4±13.3 organisms (Pm I), 92.6±30.1 organisms (Pm II) and 117.9±21.3 organisms (Pm IV)) over the two salinities. The same trend was found when focusing on abundances of juveniles (effect of salinity: F_3,24_ = 6.80, P = 0.016; effect of species: F_3,29_ = 4.30, P = 0.014). Adult abundances at first dispersal were also species-specific (F_3,24_ = 5.7, P = 0.0041), with significant differences between Pm III (6.1±2.3 adults) compared with Pm I and Pm IV (resp. 57.6±11.4 and 43.5±7.5 adults), but no effect of salinity could be found (F_1,24_ = 0.41, P = 0.52) (Fig. 3b). An interaction effect between salinity and species was absent in all three cases (F_3,24_, all P>0.18). For total nematode density, significant differences within one species were found for Pm III (F_1,6_ = 7.74 , P = 0.032) and Pm IV (F_1,6_ = 14.97, P = 0.0083), with higher total abundances at a salinity of 25 (52.0±17.0 (Pm III) and 165.5±24.0 (Pm IV)) than at a salinity of 15 (4.25±2.6 (Pm III) and 70.3±5.6 (Pm IV)). No significant differences were found within one species for the adult nematode density (F_1,6_, all P>0.05).

Numbers of adults in the dispersal plate at time of first dispersal did not differ between the different salinity treatments, but did differ between the different species (ANOVA, F_3,24_ = 4.81, P = 0.0091), with higher numbers of dispersive organisms for Pm II (5.4±0.8 adults) than for Pm III (1.3±0.2 adults; P = 0.03) (Fig. 3c). One-way ANOVA's between salinities within one species did not reveal any differences (F_1,6_, all P>0.05).

### Gender effect on dispersal

**Figure 4:**
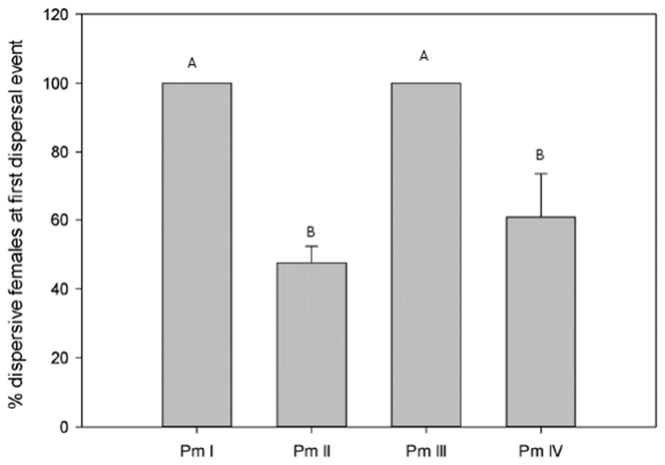
Proportion of females among dispersive adult nematodes at first dispersal (mean ± SE) for the four cryptic species of *L. marina* in the B treatment (letters indicate significant differences; p<0.05 ; n = 16).

Proportion of females among the first dispersive nematodes differed between the different cryptic species (Kruskal-Wallis, H = 11.27, P = 0.01), with consistently only females being the first dispersers in Pm I and Pm III. A more balanced ratio of dispersive females and males was found for Pm II and Pm IV (Fig. 4).

## Discussion

To date, there is only limited evidence for differential dispersal in meiofauna at the level of species [35], [56], [57], [58], particularly with respect to active dispersal. Three important characteristics explaining differential dispersal rates in benthic nematode assemblages are size [59], life history [59], [60] and vertical position inside the sediment [20]. In addition, even closely related nematode species may differentially disperse towards different food patches [61], [62].

In our experiment, active dispersal occurred in all four cryptic species of *L. marina* in less than two weeks, and significant differences between the species were observed. Pm I was the slowest disperser, taking almost one week longer to disperse than Pm III, the fastest disperser. It is unlikely that any of the above mentioned factors can explain the observed differences in time until dispersal in our study. Size differences between the cryptic species are limited [44]. The little information available on life history differences between these cryptic species (increased population growth for Pm III and Pm IV at a salinity of 15 (De Meester et al., 2011)), suggests that such differences are rather subtle, and do not clearly correlate with the dispersal differences observed here. Moreover, *L. marina* is not a true infaunal species but rather frequents patches of decomposing algae or biofilms on living algae, rendering a direct link between position in the substratum and dispersal unlikely. Finally, the food conditions were the same for all four species in the present experiments. The different dispersal ability of Pm I may, however, be related to species-specific attraction to food sources. In a preliminary experiment on the migration of these four cryptic species towards different bacterial strains, Pm I was the only species which readily moved towards *E. coli* (Derycke, unpublished data). This is surprising given the fact that lab cultures of all four species are easily maintained on *E. coli*, but these results could explain why Pm I dispersed sooner in the D treatment compared with the B and I treatments. At the same time, if Pm I has a stronger preference for *E. coli* as food than the other cryptic species, then this might also explain why Pm I generally dispersed later than the other species in the B and I treatments, but it does not explain why dispersal was equally fast when no food was available in both plates and thus no food trigger was present (N treatment) as in the D treatment. In the N and D treatment, time of first dispersal was no longer species-specific and occurred around the fourth day in all species, probably to avoid the suboptimal conditions of the inoculation plate (no food). This shows that Pm I is able to disperse faster under certain conditions and time until dispersal is not merely the result of behavioural differences in activity or motility between different cryptic species. Nematodes were also able to survive and even reproduce in plates without food, probably because they survive temporarily on energy reserves and nematodes, even after washing, still carry some bacteria from the stock cultures on their cuticles and thus spread some food even in treatments where none had been inoculated (N treatment).

No differences in time until dispersal were found between the I and B treatment, which indicates that density dependent dispersal may be important. For Pm I and Pm IV organisms disperse when densities become too high (comparable maximal densities at the same food availability in [47]), regardless of the conditions elsewhere. At the time of their dispersal, inoculation plates had already reached higher population densities as in the D and N treatments, and intraspecific interactions may be increased. Organisms will disperse to avoid crowding, even though food is still available, in agreement with results on *C. elegans* [25], [63]. In contrast with the clear density dependent effect in Pm I and Pm IV, Pm III dispersed before the fifth day in the B, I and D treatment, i. e. well before the first offspring generated in the inoculation plates became adult and density dependence could have become important. Pm II dispersed in all food treatments at a lower population density in the inoculation plate compared with the other species. This can be the consequence of higher intraspecific competition in this species. The effect of food quantity was not tested in this experiment, but we can expect that lower food availability in the inoculation plates will result in more severe intraspecific competition behaviour. Previous results already showed an inverse relationship between food availabilities and dispersal rates in a variety of invertebrates and vertebrates [25], [64], [65], [66].

**Figure 5:**
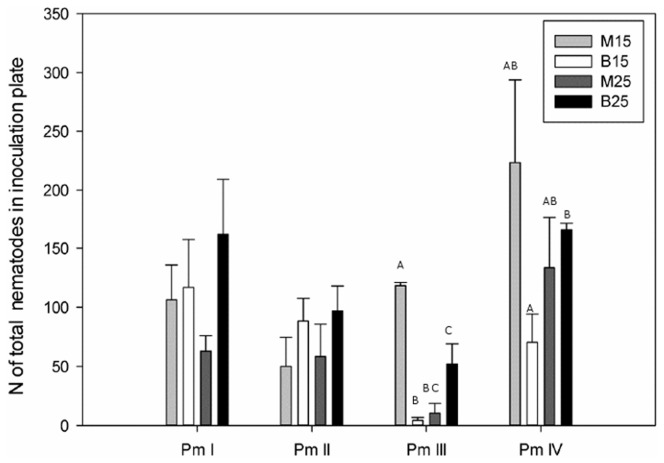
Average total number of organisms in the inoculation plate (mean ± SE) at time of first effective dispersal in plates with dispersal opportunities at two different salinities (B15: salinity of 15 ; B25: salinity of 25) compared with total number of organisms at the same time in plates without dispersal oppurtinities at the same two salinities (M15 and M25, data from De Meester et al., 2011) in four cryptic species of *L. marina* (letters above bars indicate significant differences; p<0.05; n = 16).

Besides the species-specific effect of food distribution on dispersal, salinity also had an effect on dispersal, with a generally more rapid dispersal at the lower salinity for all four cryptic species. Despite this, no significant differences were found between the two salinities within individual species, even though the average in time until dispersal over the four cryptic species was 2.4±1.0 days longer at the higher salinity. We had anticipated such a response for Pm III and Pm IV, because monospecific cultures of both species had higher growth rates at the lower salinity [4], so intraspecific competition could be expected to show up sooner at the lower salinity. However, both species showed significantly lower total densities in the inoculation plate at the time of dispersal at the lower salinity, demonstrating that the effect of salinity does not simply mirror density-dependence. We suggest that the salinity effect on dispersal may be a consequence of a different energy allocation at different salinities [67], [68], [69], [70]. When comparing total nematode densities of the present experiment with the results of a previous experiment without dispersal [4], we see that Pm III reached higher total abundances at the lower salinity in cultures where no dispersal was possible compared with the present experiment in which dispersal was possible (resp. 132.8±44.8 and 4.3±2.6 nematodes at the time of dispersal, Kruskal-Wallis test, H = 10.59, P = 0.014; Fig. 5). These results should be interpreted with caution because both experiments were not performed simultaneously, but total population densities obtained in both experiments were comparable. These results support the energy allocation hypothesis: if organisms have the chance to disperse, they will spread their energy first over dispersal, and postpone reproduction until they arrive at the new plate, which is indicated by the rapid growth in the dispersal plate (around day 6 the population abundance in the dispersal plate was higher than in the inoculation plate in all 4 replicates). These differences were not found at a salinity of 25, where Pm III showed comparable total densities in plates with and without dispersal opportunities (resp. 52.0±16.9 and 10.5±8.2 nematodes at the time of dispersal, ANOVA between two treatments, F_1,7_ = 4.86, P = 0.07; Fig. 5). However, Pm III showed a higher juvenile density in the inoculation plate in the I treatment compared with the B treatment, which could be explained by the absence of a food trigger in the dispersal plate in the first treatment, leading to more investment of energy in reproduction than in dispersal. For Pm IV no differences were found between densities in plates with and without dispersal opportunities at lower salinity (F_1,6_ = 0.07, P = 0.95; Fig. 5), so no differences in energy allocation were found for this species. The higher density in plates with dispersal opportunities at the salinity of 25 compared with the salinity of 15 could be due to differences in time until first dispersal, which was on average 2 days shorter at a salinity of 15 than of 25, although not significantly different from time until dispersal at the lower salinity (6.3±0.9 days). These differences in densities were completely due to the number of juveniles (F_1,6_ = 35.06, P = 0.0010), which could point out that at the higher salinity the second generation already started to reproduce in contrast with the population dynamics at the lower salinity. The increased population growth at a salinity of 15 in cultures without dispersal opportunities [4] had no effect on the dispersal ability of the species and dispersal occurred at both salinities at a time when no differences in total densities between the two salinities were found (Fig. 5).

The number and gender of dispersive organisms also differed between the cryptic species. The salinity experiment showed that number of adults in the dispersal plate differed between Pm II and Pm III. Moreover, Pm IV followed the same trend as Pm II, and Pm I as Pm III. This trend was also found in the food distribution experiment. Pm I and Pm III had mostly only one or two dispersive individuals at the time of first dispersal and these were always females. In the days after the first dispersal event, males also arrived in the dispersal plate, invalidating the possibility of sex-biased dispersal in these species. The fact that in Pm I and Pm III the first dispersers were always females could theoretically be a consequence of female dominance in the populations. Preliminary results showed that for Pm III populations a biased male∶female ratio exists (72.1±11% females), which could partly explain why females were the first dispersers, even though even this sex ratio should not result in 100% of the first dispersers being females. Moreover, the male∶female ratio is more balanced in populations of Pm I (53.8±6.1% females), so the fact that females were always the first dispersers clearly reflects sex-biased dispersal. This could result from fitness differences between males and females [71]. Indirect support for this hypothesis comes from the observation that females of Pm I tend to have somewhat shorter development times than males [46], [47].The dispersal in the next days could then be triggered by the first dispersers, which leave mucus tracks on which bacteria can easily grow [72], resulting in a food ‘trail’ towards the new patch. Pm II and Pm IV dispersed in most of the treatments with a higher number of organisms, with an almost perfectly balanced (1∶1) ratio males∶females. Here, it is more likely that individual rather than gender-specific differences in fitness [73] lead to specific dispersal abilities. Another possibility is that the species react differently to environmental cues or cues produced by conspecifics [63]. When no food was available in both plates (N treatment), Pm IV dispersed with significantly fewer organisms compared with the D treatment, possibly the result of the absence of a food trigger. No such differences between food treatments nor between salinity treatments were, however, found in the other species, suggesting that the effects of environment on dispersal depend on the species.

All four cryptic species showed highly efficient dispersal, the proportion of successful dispersal events exceeding 95% in all four cryptic species and under all experimental conditions (Kruskall-Wallis test for food, salinity and species: all P>0.66). The high dispersal rates observed here indicate that dispersal over short distances (10 cm) may be common in natural environments too. In natural environments dispersal will happen in a landscape mosaic [74] and not just from one location to the other as in this experiment. Organisms will thus be able to move from and to different patches in search of better spots. This can lead to dispersal over larger distances. The fact that organisms only start to disperse after a few days instead of a few hours, can indicate that dispersal comes at a cost. Costs for active dispersal are mostly considered to be loss of reserves due to increased locomotory activity [75]. Although these costs are expected to be small (only a few % of the total metabolic costs [76]), time and risk (for instance an increased predator-prey encounter probability [77]) costs should also be taken into account. That dispersal goes with a cost is shown in the N treatment for Pm III, where the dispersal plate of one of the replicates went extinct. Moreover, time until dispersal was somewhat slower, which could be the result of the absence of food and thus energy resources. This trend was not seen in the other species. Dispersal can be a selective advantage when the fitness benefits of dispersal exceed the costs of movement [78]. When local conditions become less favourable (e.g. food depletion, higher intraspecific competition, etc.), dispersal will be beneficial.

Our study demonstrates that differences in time until dispersal between very closely related nematode species exist. Dispersal is in most cases density-dependent. However, Pm III had a shorter dispersal time compared with Pm I, and dispersed well before high densities were reached in the inoculation plate. Moreover, food distribution and salinity can alter the timing of dispersal in cryptic species of *L. marina*. This response is species- and condition-specific. If active dispersal is common in natural environments, patches where species go extinct, can easily become colonised again [79], which can contribute to the resilience of populations [51]. The typical habitat of *L. marina* consists of ephemeral patches of macroalgal wrack washed ashore, and local populations are hence subject to pronounced colonization-extinction dynamics [79]. The species-specific differences in dispersal strategy can have important consequences for metapopulation and metacommunity dynamics, genetic diversity and species composition in newly establishing populations and assemblages, for instance if priority effects, where the first arriving species will have an advantage over the following species, occur [79], [80]. Clear priority effects within a single cryptic species of *L. marina* (Pm I) have been demonstrated in a field experiment, impacting the genetic structure and diversity of local populations [79]. However, we are unaware of any studies demonstrating priority effects between different nematode species. The active dispersal observed here over small distances may affect dispersal at larger scales, since it may facilitate passive dispersal as well. The differences in dispersal can also affect the response of cryptic species to competition and can help explain temporary coexistence between cryptic species. For instance, weaker competitors could be expected to disperse sooner. From the mixed-species experiment by De Meester et al. [4], however, Pm I and Pm III proved to be the stronger competitors and Pm II and Pm IV the weaker ones, so both the slowest and fastest disperser in our current experiments appear to be strong competitors. For testing this hypothesis, more information about the interaction between dispersal and other biological factors (e.g. competition) is necessary to better understand the mechanisms underlying this coexistence. In a future experiment, microcosms with dispersal opportunities, in which all four cryptic species are placed together, will be started up to record differences in time until dispersal between the cryptic species when competition between the different species is present.
